# External validation and updating of DIGIROP prediction models including parenteral nutrition for treatment-requiring retinopathy of prematurity in a Swedish national cohort

**DOI:** 10.1136/bmjophth-2026-002727

**Published:** 2026-04-02

**Authors:** Aldina Pivodic, Berg Karin, Jenny Wallander, Lois Smith, Eva Larsson, Despina Tsamadou, Karin Sandgren Hochhard, Kristina Teär Fahnehjelm, Lotta Gränse, Gerd E Holmstrom, Ann Hellström, Pia Lundgren

**Affiliations:** 1 Department of Clinical Neuroscience, Institute of Neuroscience and Physiology, Sahlgrenska Academy, University of Gothenburg, Gothenburg, Sweden; 2 Department of Ophthalmology, Sahlgrenska University Hospital, Sahlgrenska University Hospital, Region Västra Götaland, Gothenburg, Sweden; 3 The Department of Ophthalmology, Boston Children’s Hospital, Harvard Medical School, Boston, Massachusetts, USA; 4 Department of Surgical Sciences, Ophthalmology, Uppsala University, Uppsala, Sweden; 5 Department of Ophthalmology, Örebro University Hospital, Örebro, Sweden; 6 Department of Clinical Sciences, Ophthalmology, Umeå University, Umeå, Sweden; 7 Department of Clinical Neuroscience, Section for Eye and Vision, Karolinska Institute, Stockholm, Sweden; 8 Department of Pediatric Ophthalmology, St Erik Eye Hospital, Stockholm, Sweden; 9 Department of Clinical Sciences, Ophthalmology, Skåne University Hospital, Lund University, Lund, Sweden

**Keywords:** Retina

## Abstract

**Background/aims:**

This study represents an external validation with model updating of the online DIGIROP-Prescreen and DIGIROP-Screen 2.0 prediction models, incorporating ≥14 days of parenteral nutrition as an additional predictor, to identify infants requiring treatment for retinopathy of prematurity (ROP) in a contemporary Swedish cohort, estimate potential cost savings and compare DIGIROP performance with other models.

**Methods:**

Infants born in Sweden, 2021**–**2023 (n=1530) were evaluated. ROP and neonatal data were retrieved from Swedish quality registers. Sensitivity, specificity and the area under the receiver operating characteristic (AUC) curve were calculated. In the Västra Götaland Region cohort (n=245), the DIGIROP models were compared with WINROP and two Postnatal Growth and ROP (G-ROP) models. We estimated DIGIROP models’ cost-saving.

**Results:**

The mean gestational age was 27.7 (SD 2.2) weeks, birth weight 1029 (SD 317) g, 689 (45.0%) were girls and 85 (5.6%) infants received ROP treatment. For DIGIROP-Prescreen 2.0, the AUC was 0.89 (95% CI 0.86 to 0.92), sensitivity was 100% (95% CI 95.8% to 100%) and specificity 27.5% (95% CI 25.3% to 29.9%). For DIGIROP-Screen 2.0, the cumulative specificity increased from 27.7% to 67.8% between postnatal weeks 6–14, with a sensitivity of 100%. In total, ∼Int$750 000 could have been saved in screening costs in Sweden during 2021–2023 using DIGIROP models. DIGIROP-Screen at postnatal age 10 weeks had the same sensitivity but higher specificity than G-ROP. WINROP showed lower sensitivity but the highest specificity.

**Conclusion:**

DIGIROP 2.0 demonstrated high sensitivity and the most robust discrimination for treatment-requiring ROP in a contemporary Swedish cohort, compared with other models, with the potential to reduce unnecessary eye examinations and healthcare costs.

**WHAT IS ALREADY KNOWN ON THIS TOPIC**

**WHAT THIS STUDY ADDS**

- The present study demonstrates 100% sensitivity of DIGIROP 2.0 prediction models, under conditions of complete and timely screening data, for identifying infants at high risk of requiring ROP treatment in a modern national cohort, with higher specificity than the most referenced prediction models. DIGIROP 2.0 prediction can produce substantial cost savings.

**HOW THIS STUDY MIGHT AFFECT RESEARCH, PRACTICE OR POLICY**

- Using the updated DIGIROP 2.0 prediction models may save infants, parents, personnel and the healthcare system from unnecessary examinations and substantial costs without increasing the risk of visual impairment.

## Introduction

 Retinopathy of prematurity (ROP) is one of the leading causes of visual impairment and blindness in children worldwide.^1^ It can be partially prevented if infants are treated when they reach threshold criteria uncovered by screening.^2^ Screening criteria vary across countries but are primarily based on gestational age at birth (GA) and/or birth weight (BW) to identify infants who have reached the threshold for treatment-requiring ROP (TR-ROP), as determined by serial retinal examinations.^3^ Using GA and/or BW only as screening criteria and the use of dilated retinal examinations to determine if the retina has reached the point that treatment is beneficial can be very sensitive but not very specific, leading to repeated unnecessary retinal examinations of infants, as only a small percentage of screened infants actually require treatment for ROP.^4^

Swedish guidelines recommend screening of infants born before 30 weeks of GA and more mature severely ill preterm infants. In Sweden, ROP screening begins no earlier than 31 weeks of postmenstrual age (PMA) or 6 weeks after birth, whichever is later. Screenings with dilated retinal examinations are repeated based on disease severity until TR-ROP develops or the retina is fully vascularised.^5^ Eye examinations are stressful for infants and require extensive resources of ophthalmic healthcare.^6^ Prediction models to assess an infant’s individual risk for TR-ROP allow clinicians to focus on retinal examination screening of higher-risk infants, limit unnecessary examinations, improve infants’ well-being and optimise resources.

The first models (WINROP)^7 8^ proposed to improve the specificity of predicting which infants will develop TR-ROP, using GA and BW as well as the additional risk parameters insulin-like growth factor 1 (IGF-1) levels, postnatal increases in body weight (reflecting IGF-1 levels) and ROP status. Several later models used GA, BW and postnatal growth, such as Postnatal Growth and ROP (G-ROP), Colorado-ROP, Children’s Hospital of Philadelphia-ROP and Omaha-ROP. Some models also incorporated medical conditions, history of hydrocephalus, blood transfusion or prolonged oxygen supplementation.^9^,^13^ Pivodic *et al* have developed prediction models to be used sequentially for TR-ROP applicable to infants born at GA 24–30 weeks called DIGIROP-Birth and DIGIROP-Screen.^14 15^ DIGIROP-Birth predicts the individual infant’s risk of developing TR-ROP based on sex, GA at birth and BW. If DIGIROP-Birth recommends further screening, the second model, DIGIROP-Screen, can be used. It includes the timing of ROP development to assess TR-ROP risk during screening from 6 to 14 weeks’ postnatal age (PNA), potentially recommending release from further screening while screening. Both of the first iteration of DIGIROP models have been validated internally and externally in Germany, USA and Greece and are ongoing in Taiwan and Denmark.^14^,^24^ To further improve sensitivity and specificity, Pivodic *et al* then created two new models (DIGIROP-Prescreen 2.0 and DIGIROP-Screen 2.0) that integrate a comorbidity factor, in which a cut-off of 14 days or more of parenteral nutrition (PN) at PNA 4 weeks demonstrated the most significant predictive capability.^25^

This study aimed to perform an external validation with model updating of the updated DIGIROP-Prescreen and DIGIROP-Screen 2.0 models, incorporating days of PN, in a national contemporary cohort of all ROP-screened infants born in Sweden from 2021 to 2023.

## Methods

### Study population

Infants screened for ROP in Sweden, born between 1 January 2021 and 31 December 2023 (n=1534), were eligible for inclusion in the study. Four infants with missing BW data and no ROP treatment were excluded, resulting in a final cohort of 1530 infants for this validation study. Data on GA, BW, sex, ROP, including maximum ROP stage, ROP treatment (PNA age at treatment), screening examination dates, ROP detection at screening examination (yes/no) and the number of screening examinations, were retrieved from the Swedish national ROP registry (SWEDROP). Data on days of PN were retrieved from the Swedish Neonatal Quality Register, with a cut-off of 14 days during first four PNA weeks were applied. In addition, DIGIROP 2.0 models were compared with WINROP and G-ROP using the Västra Götaland Region (VGR) subset of 268 infants. 23 of these infants were excluded because longitudinal weight data were missing, preventing WINROP and G-ROP calculations. The final comparison cohort included 245 infants, of whom 25 received ROP treatment. To estimate cost savings, data from all 268 infants in the VGR cohort were used.

Neither the funders, the patient nor the public was involved in the design, conduct and reporting of the study.

### Study procedures

In the national cohort, ROP examinations were performed using RetCam and/or indirect ophthalmoscopy; in VGR, a majority of the examinations were performed using RetCam. ROP classification and treatment was performed according to international guidelines.^26 27^

### Data requirements for prediction models

*DIGIROP-Prescreen 2.0:* GA, BW, sex and cut-off 14 days of PN or more during the first four postnatal weeks. Missing data were handled as an Unknown category supported by the model. The inclusion of ≥14 days of PN represents a model updating step, while all other predictors and model structure remained unchanged from the previously published DIGIROP models.

*DIGIROP-Screen 2.0:* Predicted estimates for TR-ROP from DIGIROP-Prescreen, current screening date, occurrence of any stage of ROP (yes/no) at screening examination (date for first diagnosis of any stage of ROP).

*WINROP:* GA (<32 weeks), BW, sex and weekly weight gain up to PMA week 36.

*G-ROP:* GA (<28 weeks) or BW (<1051 g), weight gain day 10–19 (<120 g), day 20–29 (<180 g), day 30–39 (<170 g) and presence of hydrocephalus or not.

*G-ROP 180 g:* GA (<28 weeks) or BW (<1051 g), weight gain day 10–19 (<180 g), day 20–29 (<180 g), day 30–39 (<180 g) and presence of hydrocephalus or not.

### Cost estimations of screening

In the VGR subset (n=268), exact dates for all ROP screening examinations were available. Using the continuous results from DIGIROP-Prescreen and DIGIROP-Screen 2.0 over PNA up to 14 weeks, the number of potentially avoidable visits was calculated. According to the economic department of the Sahlgrenska University Hospital, the average hospital staff cost per screening examination during 2021–2023 was Kr3684, which corresponds to the cost of Int$439 per screening examination. The conversion was conducted using an index for local salary and price change in healthcare, adjusted for quality change but excluding medications, (3.7% increase from 2021 to 2022)^28^ and thereafter converted using purchasing power parities^29^ from SEK to Int$ (conversion rate in 2022: Kr8.4=Int$1), according to the recommended methodology for non-tradable resources.^30^ To convert the costs from VGR to national Swedish costs for 2021–2023, the factor 1530/268 (the number of infants screened in Sweden divided by the number of infants screened in VGR during 2021–2023) was applied to the VGR cohort costs.

### Statistical analysis

Sensitivity, specificity, cumulative specificity over PNAs 6–12 weeks models, negative predictive value, positive predictive value, accuracy and area under the receiver operating characteristic curve (AUC) were described as predictive measures for the models, all with 95% CI.

Descriptively, continuous variables were presented as mean±SD, median and range, and categorical variables were presented as frequencies and percentages.

For testing between groups, Fisher’s exact test was used for dichotomous variables, the Mantel-Haenszel χ² trend test for ordered categorical variables and the Mann-Whitney U-test for continuous variables. The sample size was determined by available national registry data. The number of outcome events was considered sufficient to provide precise estimates of model discrimination and sensitivity in this external validation study.

All tests were two-tailed. P values <0.05 were considered significant. All analyses were performed using SAS software V.9.4 (SAS Institute, Cary, North Carolina, USA).

This study is reported in accordance with the TRIPOD (Transparent Reporting of a multivariable prediction model for Individual Prognosis Or Diagnosis) statement for external validation studies.

## Results

### Study population

Of the 1530 infants included in the study, 689 (45.0%) were girls. There were significant differences in GA and BW between the infants not requiring treatment and those needing treatment, 27.8 (SD 2.1) weeks versus 24.7 (SD 1.6) weeks, (p<0.0001) and 1050 (SD 311) g versus 674 (SD 172) g, (p<0.0001). Any ROP was diagnosed in 577 (37.7%) infants, and 85 (5.6%) of the infants in the study received ROP treatment. Median PNA at the first ROP treatment was 13.1 weeks (range 5.7–31.1). Infants not requiring treatment had a significantly lower median number of screening examinations of 6.2 (SD 4.7), compared with 19.3 (SD 6.6) examinations in the group of infants that required treatment (p<0.0001), table 1.

**Table 1:** Infants’ characteristics for ROP treated and not treated infants (SWEDROP validation cohort 2021–2023)

	No ROP treatment n=1445	ROP treatment n=85	P value
Sex			0.50
Boy	791 (54.7)	50 (58.8)	
Girl	654 (45.3)	35 (41.2)	
Gestational age at birth (weeks)	27.8±2.1 28.1 (22.0 to 37.3) n=1445	24.7±1.6 24.7 (22.0 to 29.9) n=85	<0.0001
Birth weight (g)	1050±311 1030 (401 to 2950) n=1445	674±1 72 647 (382 to 1172) n=85	<0.0001
Birth weight SDS	−1.2±1.5 −0.9 (−7.9 to 2.7) n=1387	−1.2±1.4 −1.1 (−5.5 to 1.5) n=62	0.60
Maximum ROP stage			<0.0001
No ROP	953 (66.0)	0 (0.0)	
ROP stage 1	136 (9.4)	0 (0.0)	
ROP stage 2	220 (15.2)	1 (1.2)	
ROP stage 3	136 (9.4)	76 (89.4)	
ROP stage 4A	0 (0.0)	5 (5.9)	
ROP stage 4B	0 (0.0)	2 (2.4)	
ROP stage 5	0 (0.0)	1 (1.2)	
Postnatal age at first ROP diagnosis (weeks)	8.7±2.5 8.3 (4.4 to 22.9) n=492	9.2±2.0 9.3 (5.3 to 14.7) n=85	0.0095
Postnatal age at first ROP treatment (weeks)		13.4±3.6 13.1 (5.7 to 31.1) n=85	
Number of ROP screening examinations	6.2±4.7 4 (1 to 42) n=1445	19.3±6.6 19 (5 to 46) n=85	<0.0001
Parenteral nutrition duration first 4 weeks (days)			<0.0001
<14 days	897 (62.1)	24 (28.2)	
≥14 days	369 (25.5)	59 (69.4)	
Unknown	179 (12.4)	2 (2.4)	
DIGIROP-Prescreen 2.0 risk estimate	0.060±0.108 0.010 (0.000 to 0.720) n=1445	0.268±0.168 0.234 (0.003 to 0.721) n=85	<0.0001
DIGIROP-Prescreen 2.0 decision support tool			<0.0001
No screening	398 (27.5)	0 (0.0)	
Screening	1047 (72.5)	85 (100.0)	

Data are presented as mean±SD, median (range) and number of observations, or number (percentage).

For the test between two groups, Fisher’s exact test was used for dichotomous variables, Mantel-Haenszel χ² test for ordered categorical variables and Mann-Whitney U-test for continuous variables.

ROP, retinopathy of prematurity; SDS, standard deviation score; SWEDROP, Swedish national ROP registry.

### PN duration and ROP

A total of 428 infants (28.0%) received PN for ≥14 days. Among the ROP-treated infants, 59/85 (69.4%) had PN ≥14 days, whereas among the non-treated infants, 369/1445 (25.5%) had PN ≥14 days, p<0.0001.

### Validation of DIGIROP-Prescreen 2.0

**Figure 1:**
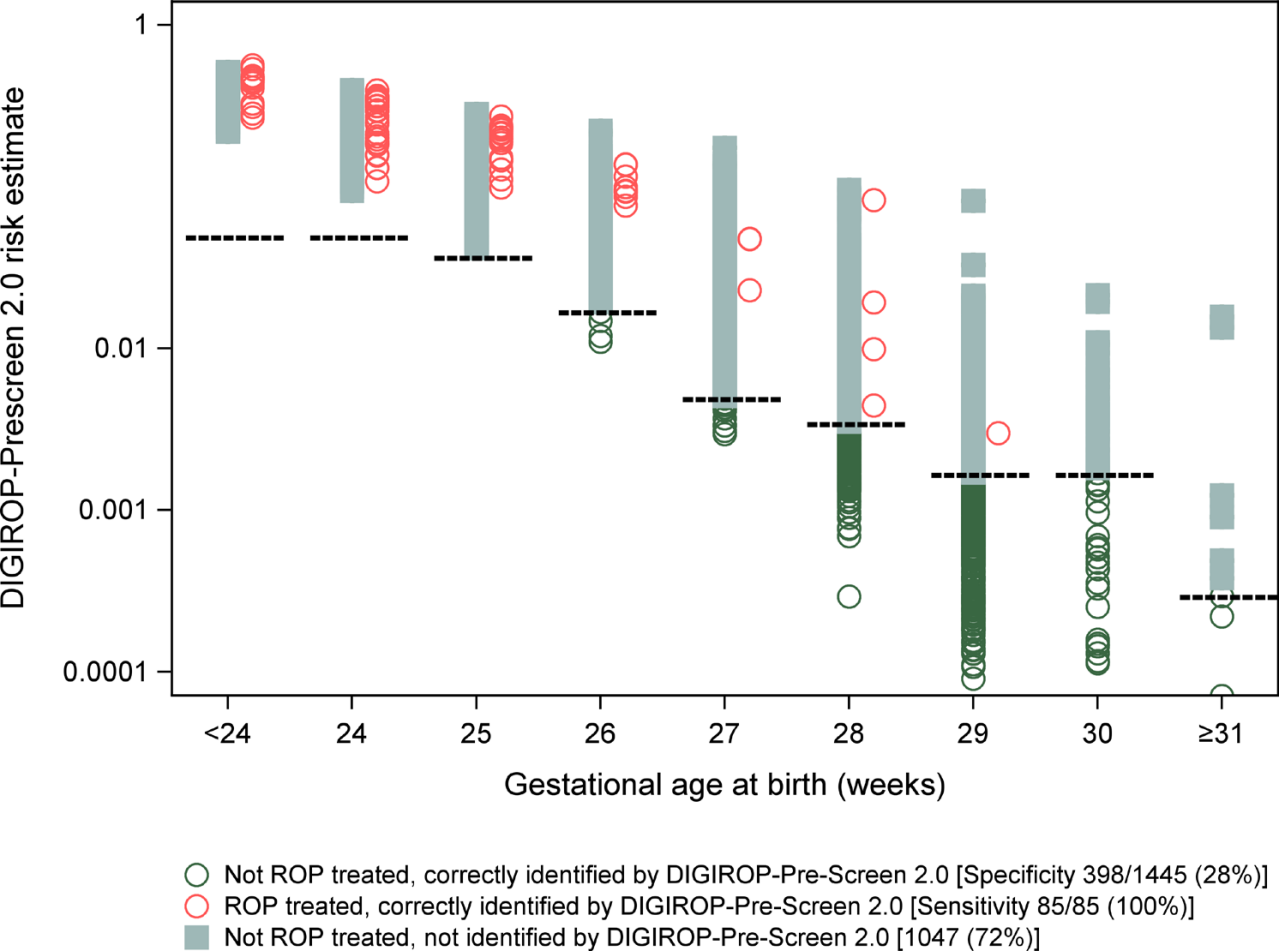
DIGIROP-Prescreen 2.0 risk estimates and outcome from decision support tool (SWEDROP validation cohort 2021–2023). ROP, retinopathy of prematurity; SWEDROP, Swedish national ROP registry.

For DIGIROP-Prescreen 2.0 alone, the sensitivity in the national Swedish cohort was 100% (95% CI 95.8% to 100%), and the specificity was 27.5% (95% CI 25.3% to 29.9%) (figure 1). The calibration plot also indicated a well-calibrated model for this validation cohort (online supplemental figure 1). This confirms that the updated DIGIROP-Prescreen 2.0 model remained well calibrated when externally validated in an independent national cohort.

In the national cohort, 102 of 296 infants (34.5%) born at 28 weeks’ GA could have been discharged from screening if evaluated at PNA 4 weeks, and 230 of 384 (59.9%) born at 29 weeks’ GA. No infants born at 25 weeks’ GA or less could have been discharged from screening (online supplemental figure 2).

### Validation of DIGIROP-Screen 2.0

The DIGIROP-Screen 2.0 model demonstrated 100% sensitivity throughout PNA weeks 6–14, under conditions of complete and timely screening data. One infant was excluded from the sensitivity evaluations at week 9 because, by this time, this patient had missed 3 weeks of screening examinations and no data on the first sign of ROP were available in the database; this infant was identified as needing screening with DIGIROP-Prescreen 2.0. The cumulative specificity increased from 27.7% (95% CI 25.4% to 30.1%) at PNA 6 weeks to 67.8% (95% CI 65.3% to 70.2%) at PNA 14 weeks (table 2). Cumulative specificity in relation to GA at birth is presented in online supplemental figure 3. The AUC ranged between 0.89–0.93.

**Table 2:** Sensitivity, specificity, cumulative specificity, PPV, NPV, model accuracy, AUC and 95% CI for DIGIROP-Prescreen 2.0 and DIGIROP-Screen 2.0 (SWEDROP validation cohort 2021–2023)

Model and time point	Sensitivity		Specificity	Cumulative specificity	PPV	NPV	Model accuracy	AUC
	n/N	% (95% CI)	% (95% CI)	% (95% CI)	% (95% CI)	% (95% CI)	% (95% CI)	AUC (95% CI)
**DIGIROP-Prescreen 2.0**	85/85	100.0 (95.8 to 100.0)	27.5 (25.3 to 29.9)	27.5 (25.3 to 29.9)	7.5 (6.0 to 9.2)	100.0 (99.1 to 100.0)	31.6 (29.2 to 34.0)	0.89 (0.86 to 0.92)
**DIGIROP-Screen 2.0 PNA 6 w**	84/84	100.0 (95.7 to 100.0)	23.1 (21.0 to 25.4)	27.7 (25.4 to 30.1)	7.0 (5.6 to 8.6)	100.0 (98.9 to 100.0)	27.3 (25.1 to 29.6)	0.89 (0.87 to 0.92)
**DIGIROP-Screen 2.0 PNA 7** w	84/84	100.0 (95.7 to 100.0)	22.6 (20.4 to 24.8)	27.9 (25.6 to 30.3)	7.0 (5.6 to 8.6)	100.0 (98.9 to 100.0)	26.8 (24.6 to 29.1)	0.90 (0.87 to 0.92)
**DIGIROP-Screen 2.0 PNA 8** **w**	83/83	100.0 (95.7 to 100.0)	30.5 (28.2 to 33.0)	36.3 (33.8 to 38.8)	7.6 (6.1 to 9.4)	100.0 (99.2 to 100.0)	34.3 (31.9 to 36.7)	0.90 (0.87 to 0.92)
**DIGIROP-Screen 2.0 PNA 9 w**	80/81	98.8 (93.3 to 100.0)	41.2 (38.6 to 43.8)	44.3 (41.7 to 46.9)	8.6 (6.9 to 10.6)	99.8 (99.1 to 100.0)	44.2 (41.7 to 46.8)	0.90 (0.88 to 0.93)
**DIGIROP-Screen 2.0 PNA 10 w**	79/79	100.0 (95.4 to 100.0)	44.2 (41.6 to 46.8)	47.7 (45.1 to 50.3)	8.9 (7.1 to 11.0)	100.0 (99.4 to 100.0)	47.0 (44.5 to 49.6)	0.92 (0.90 to 0.94)
**DIGIROP-Screen 2.0 PNA 11w**	70/70	100.0 (94.9 to 100.0)	54.0 (51.4 to 56.6)	58.7 (56.1 to 61.2)	9.5 (7.5 to 11.9)	100.0 (99.5 to 100.0)	56.2 (53.6 to 58.7)	0.93 (0.91 to 0.95)
**DIGIROP-Screen 2.0 PNA 12w**	56/56	100.0 (93.6 to 100.0)	51.7 (49.1 to 54.3)	59.2 (56.6 to 61.7)	7.4 (5.7 to 9.5)	100.0 (99.5 to 100.0)	53.5 (50.9 to 56.0)	0.93 (0.91 to 0.95)
**DIGIROP-Screen 2.0 PNA 13w**	44/44	100.0 (92.0 to 100.0)	64.7 (62.2 to 67.1)	67.8 (65.3 to 70.2)	7.9 (5.8 to 10.5)	100.0 (99.6 to 100.0)	65.7 (63.3 to 68.1)	0.93 (0.91 to 0.95)
**DIGIROP-Screen 2.0 PNA 14w**	32/32	100.0 (89.1 to 100.0)	64.0 (61.5 to 66.5)	67.8 (65.3 to 70.2)	5.8 (4.0 to 8.1)	100.0 (99.6 to 100.0)	64.8 (62.3 to 67.3)	0.93 (0.91 to 0.96)

One infant was missed and was identified as needing treatment using DIGIROP-Screen 2.0 at PNA 9 weeks due to missed screening examinations during several weeks, so that the first sign of ROP was not correctly identified in the database.

Cumulative specificity represents the cumulative percentage of infants who could be correctly released from screening at each PNA week among those who will not require ROP treatment.

AUC, area under the receiver operating characteristic; NPV, negative predictive value; PNA, postnatal age; PPV, positive predictive value; ROC, receiver operating characteristic; ROP, retinopathy of prematurity; SWEDROP, Swedish national ROP registry; w, weeks.

### DIGIROP 2.0 models compared with WINROP and G-ROP models

For validation of the additional prediction models, WINROP, G-ROP and G-ROP 180 G, only the VGR cohort was used as it had available longitudinal weight data needed for G-ROP calculations. This cohort comprised 245 infants, of whom 24 were treated for severe ROP.

**Figure 2:**
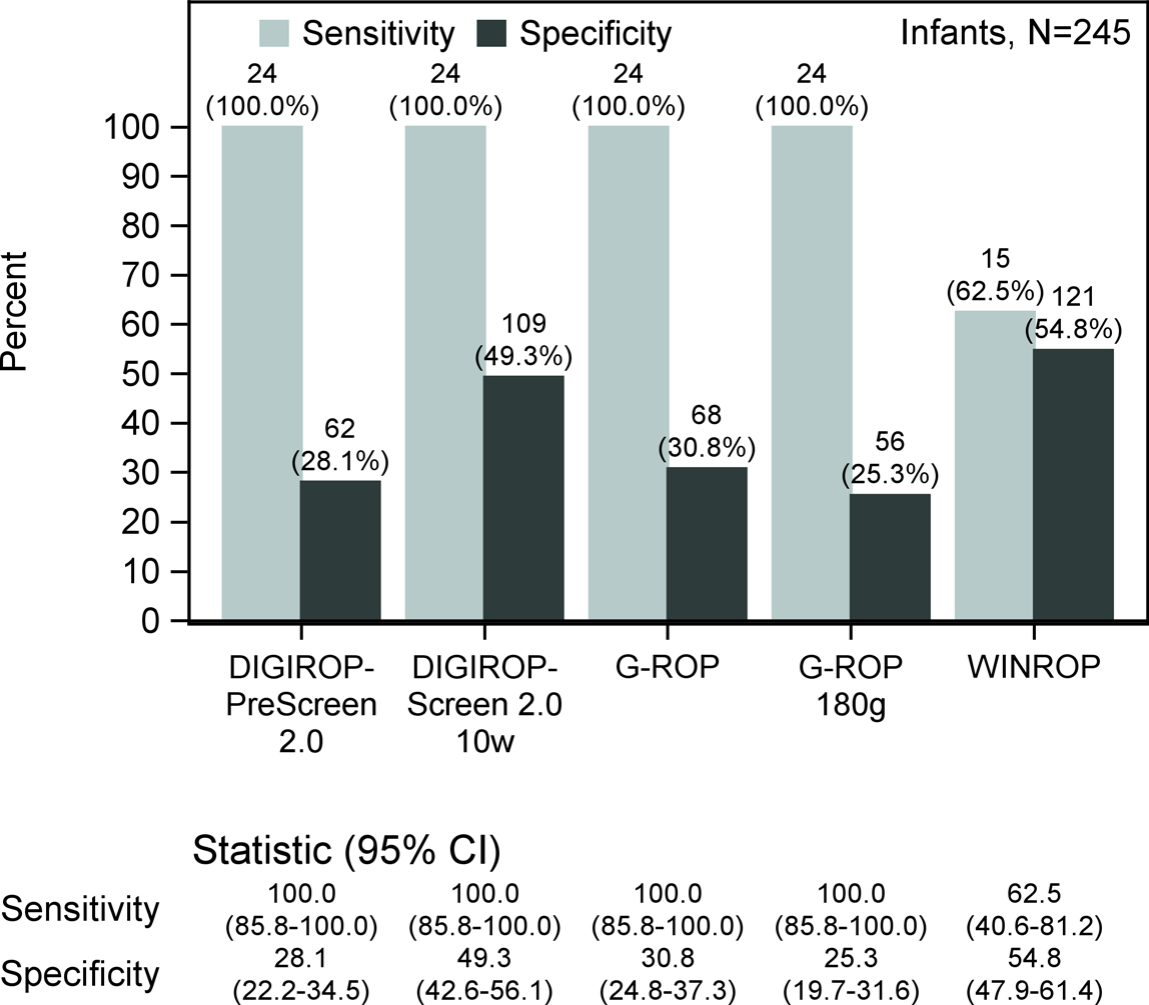
DIGIROP 2.0 models versus G-ROP versus WINROP (VGR validation subcohort 2021–2023). G-ROP, growth and retinopathy of prematurity; VGR, Västra Götaland Region.

Sensitivity was 100% for the DIGIROP 2.0 and G-ROP models but only 62.5% for the WINROP model. Specificity ranged from 25.3% to 30.8% for the G-ROP models and 28.1% to 63.3% for the DIGIROP-Prescreen and Screen 2.0 models, with 49.3% at 10 weeks PNA. The specificity of the WINROP model was 54.8% (figure 2).

### Cost estimations of screening

**Figure 3:**
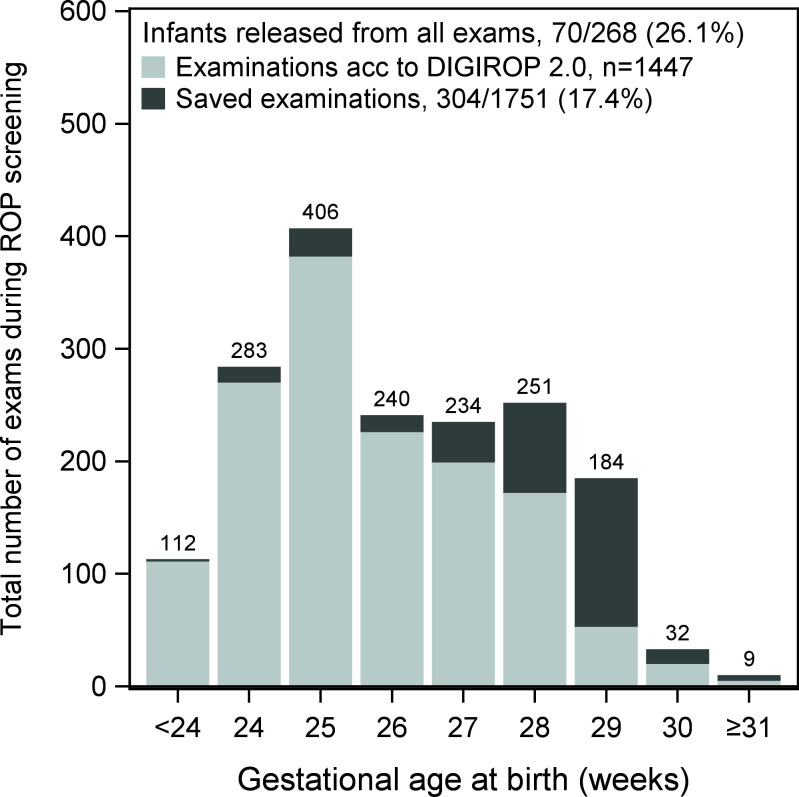
Saved number of examinations among total per gestational age using DIGIROP 2.0 models (VGR validation subcohort 2021–2023). ROP, retinopathy of prematurity; VGR, Västra Götaland Region.

In the VGR cohort, 268 infants were screened, and 1751 examinations were conducted. Using DIGIROP-Prescreen 2.0 70/268 (26.1%) infants could be released from all examinations, corresponding to a total of 304/1751 (17.4%), examinations that could be saved (figure 3). According to our economic department at VGR, calculating the savings in screening costs would have resulted in ∼Int$135 000 saved for VGR during 2021–2023. Extrapolating these results to the full Swedish population, a total of ∼Int$750 000 could have been saved during this 3-year period.

## Discussion

### Main findings

In this validation study, DIGIROP-Prescreen 2.0 and DIGIROP-Screen 2.0, which include days ≥14 of PN as a risk factor, correctly identified all infants later requiring treatment for ROP in a Swedish national cohort born between 2021–2023. Among low-risk infants, DIGIROP-Prescreen 2.0 safely recommended that 27.5% could forego screening, while DIGIROP-Screen 2.0 suggested that screening could be discontinued earlier for a further 40.3%. Using the DIGIROP 2.0 models could have potentially prevented an estimated 1790 examinations, leading to total savings of approximately Int$750 000 during this 3-year period in Sweden. For the involved infants and their families, avoiding unnecessary ROP screening reduces procedural risks and stress and supports more stable care routines, potentially benefiting early neurodevelopment and decreasing parental anxiety.

### Shortage of ROP screening and feasibility of DIGIROP 2.0

Preterm infant survival is increasing globally, and ROP is now the leading cause of preventable childhood blindness, with an estimated 20 000 infants affected each year.^31^ Screening remains limited, particularly in low- and middle-income countries, where screening and treatment programmes are often limited or absent. A global survey reported that 14 countries lack ROP screening and data are unavailable for 49 others, highlighting major coverage gaps.^3 27^ ROP screening is resource-intensive, requiring specialised expertise and equipment, such as wide-field retinal cameras, and the shortage of trained paediatric ophthalmologists exacerbates these challenges.^32^ Telemedicine and artificial intelligence-assisted screening offer potential solutions to the workforce shortage, but they are not yet widely implemented and do not eliminate the need for screening, as they mainly help manage workload.^32 33^

The DIGIROP 2.0 models, with a user-friendly interface (www.digirop.com) and reliance on simple clinical parameters, offer practical tools for managing ROP screening in neonatal care. Within the first four postnatal weeks, DIGIROP-Prescreen 2.0 guides whether an infant requires ROP screening using baseline data, including days of PN. DIGIROP-Screen 2.0 is unique in incorporating screening outcomes to update the infant’s risk of severe ROP after every eye examination.

### Importance of sensitivity in prediction models

ROP prediction models must prioritise high sensitivity, as missing an infant at risk could lead to blindness and lifelong disability. In this study, the DIGIROP 2.0 models demonstrated 100% sensitivity, under conditions of complete and timely screening data, correctly identifying all 85 infants who developed TR-ROP. DIGIROP-Prescreen 2.0 showed a neonatal specificity of 27.5%, increasing cumulatively to 67.8% at 14 weeks PNA using DIGIROP screen 2.0. In the VGR cohort, specificity ranged from 28.1% to 49.3% for early timepoints up to 10 weeks PNA, while sensitivity remained 100%. By comparison, G-ROP models had 100% sensitivity and 25.3–30.8% specificity, and WINROP achieved 62.5% sensitivity and 54.8% specificity. Thus, the DIGIROP 2.0 models offered superior specificity without compromising sensitivity.

An advantage of the original DIGIROP models was the ease of access to predictive data for the screening ophthalmologist. However, seriously ill infants were considered to need screening irrespective of the first DIGIROP results. To prevent these infants from being missed, the updated DIGIROP, including a comorbidity factor, PN days, was added. The updated DIGIROP 2.0 models require additional information on PN days, which may make their daily clinical use somewhat more difficult. However, the benefit of not excluding infants, due to GA or illness, is likely to outweigh the disadvantages.

### Reduced screening costs

DIGIROP-Prescreen 2.0 safely recommended that 27.5% of infants did not need screening among those not requiring ROP treatment, and DIGIROP-Screen 2.0 recommended that screening could be discontinued earlier than traditional screening procedures for an additional 40.3% (online supplemental figure 3). If the DIGIROP 2.0 models had been applied, 314 (17.4%) of screening examinations could have been avoided. Apart from sparing these infants the discomfort and stress of the examinations, this would also save resources, including ophthalmologic healthcare staff and costs. Given the number of potentially saved screening examinations, this would have saved costs of Int$750 000 during the 3-year period in Sweden. However, screening costs vary. In a meta-analysis by Gyllensten *et al* including both high- and low-income countries, mean costs per screening examination ranged between US$106–250 in high-income countries.^34^

### Importance of validation before implementation in different settings

This study presents the first validation of the DIGIROP-Prescreen and DIGIROP-Screen 2.0 models. The DIGIROP 2.0 models, like all predictive models, must be validated within the clinical environments in which they are intended to be implemented. In many low- and middle-income countries, infants with higher GA and BW remain at substantial risk of severe ROP, largely due to differences in neonatal care practices.^1 24 35^ Moreover, variations in the clinical use of PN should be considered, as this indicator of postnatal morbidity may not be applicable or interpreted consistently across settings.

### Strengths and limitations

The validation of the DIGIROP 2.0 models in this contemporary cohort is supported by robust data from the SWEDROP registry (2021–2023), with only 4 of 1534 infants excluded due to missing information. Another strength of the study is the comparison of DIGIROP with several established ROP prediction models using the same cohort, allowing for a comprehensive assessment of its relative performance.

The study has limitations. Registry data may contain occasional recording or editing errors. Furthermore, because the Swedish population is relatively homogeneous in terms of ethnicity, neonatal care provision and socio-economic conditions, the DIGIROP 2.0 models require thorough validation in more diverse populations and healthcare settings before implementation in other settings. Finally, the use of retrospective data represents an inherent limitation of the analysis.

## Conclusions

DIGIROP-Prescreen 2.0 and DIGIROP-Screen 2.0 prediction models demonstrated 100% sensitivity, under conditions of complete and timely screening data, and superior specificity to WINROP and G-ROP in this contemporary Swedish cohort. Implementation could significantly reduce the screening burden, lowering healthcare costs and reducing infant distress. While these findings support the models’ use in Sweden, validation in other populations is recommended before implementation.

## Supplementary material

10.1136/bmjophth-2026-002727 online supplemental figure 1

10.1136/bmjophth-2026-002727 online supplemental figure 2

10.1136/bmjophth-2026-002727 online supplemental figure 3

## Data Availability

No data are available.
