# Supplementary material for: External validation and updating of DIGIROP prediction models including parenteral nutrition for treatment-requiring retinopathy of prematurity in a Swedish national cohort
Source: BMJ Open Ophthalmol. 2026 Apr 2;11(2):e002727. doi: 10.1136/bmjophth-2026-002727 (PMC13052817; doi:10.1136/bmjophth-2026-002727)

Supplemental Figure 1. Calibration plot for DIGIROP-Pre-Screen risk estimates (SWEDROP validation cohort 2021-2023).

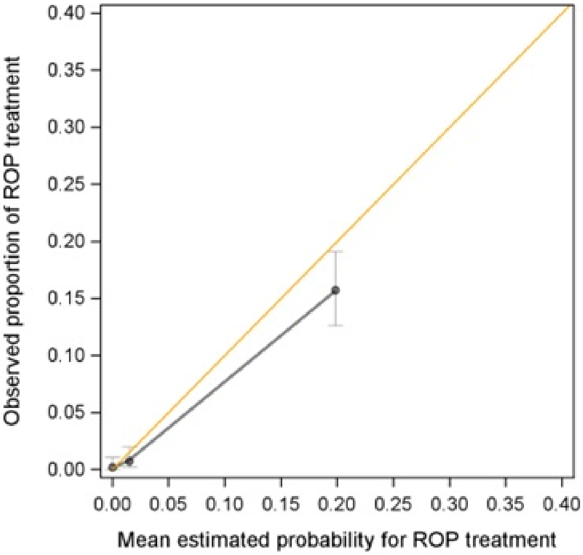

Supplement: online supplemental figure 1 [file bmjophth-11-2-s001.pdf]
